# Supplementary material for: Are carbon emissions trading and green financial instruments synergistic? -Comprehensive quantitative research based on content analysis
Source: PLoS One. 2024 Mar 7;19(3):e0298601. doi: 10.1371/journal.pone.0298601 (PMC10919601; doi:10.1371/journal.pone.0298601)
Supplement: S1 Data — (ZIP) [file pone.0298601.s001.zip › Data/地级市Z_F工作报告.zip!/╔╜╢1⁄2/╢1⁄2╙¬╩╨/└┤╫╘╣1⁄2╓┌║┼í╛┬φ┐╦╩2╛▌═°í┐_2018.docx]

各位代表：

现在，我代表市人民政府向大会报告工作，请予审议，并请市政协委员和其他列席会议的同志提出意见。

一、2017年工作回顾

刚刚过去的一年，全市上下紧密团结在以习近平同志为核心的党中央周围，深入学习贯彻党的十八大、十八届历次全会和十九大精神，以习近平新时代中国特色社会主义思想为指导，在省委、省政府和市委的坚强领导下，开拓进取，真抓实干，经济社会保持平稳健康发展，各项工作取得新成绩。

（一）克服重重困难，经济发展逆势而上。去年，经济下行压力持续加大、原油价格持续低迷，我市长期积累的经济结构性矛盾集中显现，经济、金融运行遇到前所未有的困难。面对严峻形势，我们积极应对，每月调度经济工作、每季度召开金融工作座谈会，制定出台支持工业经济发展十条意见，强力推进招商引资，推动经济发展突破困境、行稳向好。初步预计，2017年全市实现生产总值3950亿元，增长7%左右。全力实施重点项目、政府投资项目、水气土污染治理项目、民生实事项目“四张项目单子”，扭转了投资下滑局面，增幅由负转正，达到5.8%。社会消费品零售总额增长8.8%。规模以上工业主营业务收入增长21.7%，19家企业进入山东省企业百强，23家企业入围山东省民营企业百强，12家企业入围中国企业500强，18家企业入围中国民营企业500强，均居全省前列。实现一般公共预算收入232.9亿元，增长7.9%。加强政监银企结合，一企一策化解企业资金风险问题，严厉打击恶意逃废金融债务行为，维护了良好的金融生态环境，金融机构各项贷款余额3690亿元，比年初增加180亿元。

（二）大力推动新旧动能转换，产业转型升级迈出新步伐。建立了新旧动能转换项目库，筛选论证项目377个，计划总投资4147亿元。把石化产业作为新旧动能转换的突破口，严格落实“1+N”布局规划，编制了石化产业发展规划、骨干石化企业发展规划、东营港开发区发展规划、石化产业新动能三年行动计划方案。围绕建设高端石化产业基地，全力推进东营港开发区总投资414亿元的芳烃、乙烯、己内酰胺三大高端化工项目，已经取得明显进展。新帮助6家地方炼化企业争取进口原油使用量1110万吨，累计达到3110万吨。编制了油气管网规划，开工建设油气管网4条。在全省率先开展化工生产企业“四评级一评价”，去年159家“差”评企业停产整顿，淘汰落后产能1070万吨。新完成规模企业公司制改制170家，累计达到383家，提前两年完成省定任务，去年新增挂牌企业49家。启动市县产业发展基金和明月湖基金中心建设，支持企业兼并重组、对外合作，加快产业升级。东营石化产业、广饶橡胶轮胎产业成为全省转型升级示范产业集群。我市获批创建全国首个车用汽柴油产品质量提升示范区，方圆集团被列为全国有色金属行业唯一的循环经济标准化示范单位。加快发展现代农业，实施了高端乳制品加工等20个重点项目，农业龙头企业、农民合作社、家庭农场分别发展到670家、2333家、1280家。粮食再获丰收，“渤海粮仓”科技示范工程面积达到47.2万亩。加快发展现代服务业，全域旅游示范市创建工作全面启动，170家企业完成非主营业务剥离。强化创新驱动，182个项目列入省级创新计划，新培育省级技术创新平台17家，接收引进专科以上人才1.3万人，新增马德里国际注册商标100件、中国驰名商标1件、山东名牌产品9个，高新技术产业比重达到36.1%，国家采油装备工程技术研究中心、国家石油炼制产品质检中心通过验收，获批建设中国（东营）知识产权保护中心、创建首批国家知识产权强市。县域经济竞相发展，广饶县跃居全国百强县市第39位，东营区跻身全国综合实力百强区第79位。胜利油田创新发展、改革管理迈出新步伐，120多个油地合作事项扎实推进，“四供一业”及其他企业办社会职能移交接收工作有序开展；中国石油大学、济军综合训练基地发展取得新成绩。黄河三角洲农业高新示范区加快规划编制，创新管理运行体制机制，引进了山东农业大学国际现代农业科教基地等高端科教资源，实施了一批高新技术产业项目，创新能力和示范带动作用不断增强。

（三）突出生态宜居城市和美丽乡村建设，城乡面貌显著提升。坚持大空间、大绿地、大水面、大湿地“四大”特色，完成职业学院、中心医院等12个片区控规修编，被确定为全国首批城市设计试点城市。东营河及北二路环境综合整治工程在充分准备的基础上全面启动，面对这项社会关注、群众期盼、多年想干而没有干的重大工程，全市上下思想统一、认识一致，东营区、垦利区、东营开发区及市直有关单位广大干部全力以赴、细致工作、合力攻坚，沿线单位和群众积极配合、大力支持，短短几个月的时间，拆迁任务基本完成。奥体中心、图书馆建成投用，档案馆、南一路快速路基本完工，东二路、德州路、曹州路、西一路、西二路等完成改造提升。森林湿地公园一期工程主体基本完工，部分区域对外开放。白鹭湿地公园正在编制规划。利三沟水系改造工程全面完工，成为中心城一条靓丽的带状公园；中心城新建改造公园12处、游园40处、城市绿道222公里，市民有了更多休闲健身的好去处。全市完成老旧住宅小区改造167个，提前三年超额完成省定任务。棚户区改造完工9055套，实施货币化安置4491户。新开工建设城市地下综合管廊10公里。城市精细化管理水平不断提升，全市环卫机械化作业率达到90%，城区生活垃圾处理率达到100%，深度保洁道路率达到50%。拆违治乱工作走在了全省前列，各县区各有关部门创新举措、攻坚克难，两年累计拆除治理违法建设442万平方米，违法建设治理任务基本完成，城乡环境更加整洁，不正之风得到有效遏制，社会秩序明显改善。加强公路铁路沿线环境综合治理，脏乱差现象基本消除，成为贯穿城乡的生态长廊。美丽乡村标准化建设取得新进展，提前一年超额完成农村旱厕改造三年任务；在全省率先实施天然气村村通工程，完成了928个村、16.8万户的施工任务；黄河南展区新型农村社区建设持续推进；农村社区服务设施覆盖率达到93%，村庄绿化水平大幅提升；新建改造农村公路310公里，全面完成农村公路安全生命防护工程。

（四）谋划实施重大基础设施工程，大交通建设实现新突破。全市综合交通网规划和以高速铁路站点为核心的综合枢纽控制规划基本编制完成。“天津—东营—潍坊”高铁正在勘察设计，“滨州—东营—潍坊”高铁滨州至东营段完成可研报告，淄东铁路东营段完成扩能改造，黄大铁路建设顺利推进。东营港10万吨级航道、东营港疏港铁路开工建设，25万吨级单点系泊工程前期工作加快推进。广利港防波堤延伸、南防波挡沙堤建成投用，实现了开港通航。胜利机场航线增至16条，跑道延长工程建成投用，国产ARJ21飞机成功试飞。河广大道进入PPP采购程序，长深高速广饶至高青段、庐山路南延等工程开工，S507董集至利津黄河大桥段改建工程具备开工条件，河广大道、南二路西延两座黄河大桥获黄委批复同意建设。黄水东调应急工程东营段加快推进，对广南水库进行了衬砌改造，建设引水干渠98公里、输水管道8.5公里，已经达到向胶东地区送水的条件。

（五）深化改革开放，发展动力不断增强。完成了211项重点改革任务。深化“放管服”改革，调整市级行政权力事项270项，公布第一批“零跑腿”和“只跑一次”事项290项，“双随机一公开”监管全面推行。深化商事制度改革，“多证合一、一照一码”登记制度全面推行。农村集体产权制度改革试点进展顺利，农村承包土地经营权、农民住房财产权抵押贷款试点扎实推进。校长职级制、“县管校聘”等教育综合改革深入实施。公立医院改革不断深化，价格补偿机制、法人治理结构改革全面完成，分级诊疗、家庭医生签约、医联体建设实现突破。对外开放持续深化，外贸进出口总值达到1260亿元，增长26%；实际到账外资15.6亿元，增长5%；新批境外投资企业23家，实际对外投资8500万美元。东营综合保税区运行良好，筹建进口肉类指定口岸获得国家质检总局批复。橡胶轮胎展、石油装备展通过国际展览业协会（UFI）认证，数量占全省的四分之一。大力开展招商引资，成立投资促进委员会和十个产业投资促进委员会，组建投资促进局，掀起新一轮招商热潮，全年引进项目270个，到位固定资产投资407亿元。加强与央企、国企战略合作，中国商飞东营试飞基地等87个合作项目加快推进。积极引进高校、科研院所，山东胜利职业学院移交地方，中科山东东营地理研究院签约落地，北航东营飞行学院、青岛科技大学资源化研究院项目顺利推进。

（六）强力推进环境保护和生态建设，环境质量明显改善。深入实施水气土污染整治专项行动，投资62亿元实施114项治理工程，市政雨污管网改造等91个工程已经完成。全面实行河长制，开展“清河行动”和入河排污口综合整治， 9个省级以上工业园区污水实现集中处理，省控河流COD、氨氮浓度分别较上年改善12.7%、34.4%，达到地表水五类标准。实施燃煤电厂（锅炉）超低排放改造和“工业绿动力”计划，改造取缔燃煤小锅炉1881台，压减煤炭消费量129万吨，清理“散乱污”企业877家，加强扬尘和挥发性有机物等治理，二氧化硫、二氧化氮、PM10、PM2.5浓度分别较上年改善26.1%、9.5%、12.3%、13.4%。全力保障配合上级环保、海洋督察，开展整治攻坚行动，解决了一大批突出环境问题。继续实施三年增绿计划，建设了一批精品亮点工程，去年新增造林21.5万亩。加强自然保护区保护治理，修复湿地9300亩。圆满完成生态保护红线、城镇开发边界、永久基本农田保护红线划定任务，土地节约集约利用水平不断提升。

（七）着力加强社会民生建设，群众获得感持续提升。实施了改造敬老院设施等11件民生实事，财政用于社保就业、医疗卫生、住房保障支出分别增长14.8%、7.1%、7.5%。城乡居民人均可支配收入分别达到44794元、16247元，分别提高3214元、1248元。坚持精准扶贫、精准脱贫，市定标准贫困人口基本实现脱贫。直接扶持创业8233人，城镇新增就业3.6万人，失业人员实现再就业3.1万人。城市低保标准提高到每月570元以上，农村低保标准提高到每年4500元，社会保障水平居全省前列。各级各类教育协调发展，新建、改扩建中小学校37所、幼儿园49所，全市一半以上的公办中小学开展了小班化教学试点。医疗卫生事业健康发展，市人民医院急诊急救中心暨内科综合病房楼投用、获鲁班奖，国家卫生城市通过复检。创建国家公共文化服务体系示范区在中期督查中取得优异成绩，成功举办黄河口国际马拉松赛、公路自行车赛。全国文明城市通过复审。严格落实安全生产责任，深入开展“大快严”集中行动和化工产业安全生产攻坚治理行动，强化监管执法，大力整治非法违法行为和安全隐患，安全生产形势总体稳定。省级食品安全城市创建进展顺利。认真落实领导包案和公开接访制度，解决了一批群众反映的突出问题。加强社会治安综合治理，深入开展“两打一整治”专项行动，群众安全感明显提升。圆满完成党的十九大安保维稳任务。

双拥工作深入开展，退役士兵安置和权益保障政策全面落实。国防动员、人民防空、民兵预备役建设不断加强，人口计生、慈善救助、残疾人、养老、老龄事业加快发展，统计、价格、史志、气象、地震、机关事务管理工作取得新成效，民族宗教、外事侨务、对台工作实现新进步，工青妇组织发挥了重要作用，中央、省驻东营单位为地方发展作出了重要贡献。

各位代表！过去的一年极不平凡，是东营遇到困难最多、面临压力最大、发展最为复杂的一年。面对困难和挑战，全市各级干部特别是基层干部，顶住压力、勇于担当，社会各界人士和广大群众团结一致、攻坚克难，圆满完成了各项工作任务，充分展示了新时代东营人民良好的精神风貌。过去一年取得的成绩，关键在党的领导，归功于东营人民。在此，我代表市政府，向付出辛勤劳动的广大干部群众，向积极支持政府工作的人大代表、政协委员和社会各界人士，表示崇高的敬意和衷心的感谢！

在充分肯定成绩的同时，我们也清醒地认识到我市发展中还存在不少困难和问题：国内外经济形势错综复杂，部分企业资金链紧张、经营困难，投资拉动后劲不足，稳增长、防风险压力依然很大；经济结构性矛盾仍然突出，创新能力不强，产业转型升级任重道远；改善环境质量的任务艰巨，安全生产压力巨大；发展不平衡不充分的问题比较突出，民生领域仍有不少短板；政府自身建设存在薄弱环节，政务服务环境有待进一步优化。对此，我们将坚持问题导向，着力加以解决。

二、2018年工作总体要求

2018年是全面贯彻党的十九大精神的开局之年，是改革开放40周年，是决胜全面建成小康社会、实施“十三五”规划承上启下的关键一年。做好今年的政府工作，意义重大而深远。

今年政府工作的总体要求是：全面贯彻落实党的十九大精神，以习近平新时代中国特色社会主义思想为指导，坚持稳中求进工作总基调，坚持新发展理念，紧紧围绕统筹推进“五位一体”总体布局和协调推进“四个全面”战略布局，按照高质量发展要求，以推进供给侧结构性改革为主线，锁定在新旧动能转换中走在前列“一个目标定位”，突出资源型城市转型、经济转型升级“两大任务”，强化改革开放创新“三大动力”，守牢安全环保、防范风险、民生保障、社会稳定“四条底线”，促进经济社会持续健康发展和民生福祉持续改善，奋力开创美丽幸福新东营建设新局面。

今年全市经济社会发展的主要预期目标是：生产总值增长7%左右，一般公共预算收入增长6.5%，城镇新增就业3.3万人，城镇登记失业率控制在3.5%以内，进出口适度增长，居民消费价格涨幅控制在3%左右。全面完成省下达的年度节能减排降碳等约束性指标。

实现上述目标要求，必须牢牢把握原则方向。一是坚持贯彻新发展理念，积极引领经济发展新常态，加快转变经济发展方式，更加注重创新发展、绿色发展，更加注重提高经济发展的质量和效益。二是坚持新旧动能转换全面提速，以石化产业为突破口，推动优势主导产业战略性调整，积极发展新兴产业，加快建设现代化经济体系。三是坚持在环境打造上持续发力，着力建设一流的人居环境、诚信环境、基础设施环境、政务服务环境、安全稳定环境，不断提升东营的影响力、吸引力和竞争力。四是坚持底线思维和问题导向，坚决打好防范化解重大风险、精准脱贫、污染防治三大攻坚战。五是坚持以人民为中心的发展思想，抓住群众最关心最直接最现实的利益问题，一件事情接着一件事情办，在发展中不断提升保障改善民生水平，让全体东营人民共享发展成果，过上美好生活。

三、2018年重点工作

（一）突出抓好新旧动能转换重大项目建设

围绕新旧动能转换，今年安排100个市级重点项目、192个市政府投资项目、97个水气土污染治理项目、10个民生实事项目。出台全市新旧动能转换五年发展规划及三年实施方案，抓好重大项目库建设，常年筛选，滚动储备，分步实施。创新招商方式，加大政策支持，完善项目协调推进和快速落地机制，积极引进一批大项目好项目。今年政府投资主要是补缺口、保续建、保重点，要多渠道筹集建设资金，探索推进PPP、EPC项目建设模式，稳妥推动基础设施等资产证券化。落实促进民间投资的政策措施，提高民间资本投资比重。严格管理政府投资项目，把工程质量放在首位，加强招投标、监理、验收等全流程监管。优化项目审批服务，对重大投资项目开通“绿色通道”，实行“联审联办”。

（二）加快工业由高速发展转向高质量发展

推动工业发展由量的扩张加快向创新发展转变。认真落实支持工业经济发展的十条意见，以创新为引领，重点抓好四大产业集群转型升级，全力培育工业新动能。石油化工产业，围绕解决“布局散、加工浅、链条短”的问题，统一思想，形成共识，严格执行化工产业“1+N”布局规划，不符合规划的项目一律不批；加快组建大型炼化集团，整合现有炼化资源，新上高附加值项目，推动石化产业从炼化环节向“高化尾”方向发展；深入开展“四评级一评价”，列入“差”等的企业一律停产整顿，不能按期完成整改的一律关停；加快原油、成品油管网建设，力争年底实现南北主体管线贯通，抓好市域高压燃气管网建设。橡胶轮胎产业，加大政策扶持力度，推动轮胎企业兼并重组、资源整合，推动产业关联企业协同发展，加强产品研发和品牌能力建设，支持企业运用马德里品牌体系建设自主品牌，着力打造3-5家进入全球轮胎行业前列的企业。有色金属产业，依托方圆集团等骨干企业，加强对外战略合作，不断延伸产业链条，加快建设国内一流的铜产业基地。石油装备产业，对标国际一流，主攻高端技术，培育高端产品，发展高端服务，加快由生产向“生产+服务”转变。围绕四大产业集群，建设“1+5”平台，每个产业组建一个产业协会，每个产业打造一个创新平台、物流平台、展销平台、检测平台、专利平台，为工业创新发展提供支撑。支持造纸、纺织、汽车及零部件等优势产业实施技术改造，开发新产品，加快提质增效。积极培育战略性新兴产业和先进制造业，围绕新能源、新材料、新医药、新一代信息技术、高端装备制造等新兴产业，实施一批重点项目，加快形成一批新的支柱产业。

突出抓好企业创新。引导企业转变发展理念，注重企业文化建设，注重科技创新，坚守实业，做强主业。支持大企业加强自主创新能力建设，引导中小企业深化产学研结合，提高核心竞争力。支持高校院所在我市设立实体科研机构，培育一批具有行业引领能力的企业研发平台。支持胜利油田孵化器建设，为油田科技人员创新创业搭建载体。实行以增加知识价值为导向的分配政策，调动广大科研人员创新创业积极性。

坚定实施人才强市战略。坚持筑巢引凤，围绕主导产业，实施产才融合计划。制定人才支撑新旧动能转换工作实施意见，全面深化人才发展体制机制改革，实行更加积极、更加开放、更加有效的人才政策。深入推进“黄河三角洲学者”等重大人才工程，深化与国家部委、大院大所、国家“千人计划”专家团队的合作，打造“双百引才”“百千万聚才”等东营人才工作品牌。高度重视企业家队伍建设，建立市政府企业家顾问团队，制定实施企业家培养三年行动方案，组织开展一系列高端对标学习和精准化专业培训。营造企业家健康成长环境，激发和保护企业家的工匠精神、创新精神、专注精神和团队精神，更好发挥企业家作用。

推动企业发展由主要依靠银行贷款加快向利用资本市场等多元化融资转变。优化间接融资，扩大直接融资规模。加快企业规范化公司制改制，建立现代企业制度，今年完成改制100家以上，提高对接资本市场的能力。针对大企业的优质板块和成长性好的中小企业、创新型企业，制定企业上市挂牌三年规划及年度计划，落实推进政策，确保年内10家企业上市挂牌。支持企业优化债务结构、有序开展市场化债转股、发展股权融资。进一步降低企业纳税、用地、用电、用水、油气物流成本，让企业轻装上阵。

建好用好产业基金。加快设立总规模500亿元的石化产业、油服产业、企业重组、企业并购上市4支市级产业基金。尽快建成明月湖基金中心，落实优惠政策，吸引国内外优秀基金管理团队落户东营。大力引进社会基金，建立标的项目库，实行产业基金运用目标责任制，支持企业新上大项目好项目，加快产业转型升级。

（三）实施乡村振兴战略

加快推进现代农业产业园建设。选择基础条件好、产业优势突出、示范带动能力强的区域，规划建设一批市级现代农业产业园，积极做好省级产业园创建工作。以现代农业产业园为平台，集聚土地、资本、科技、人才、信息等现代要素，形成产加销、贸工农一体化的农业全产业链。调整完善畜牧园区发展规划，优先安置禁养区关闭搬迁户，推进畜牧业转型升级。实施“优质粮食工程”，保障粮食安全和粮油产品有效供给。鼓励农业龙头企业通过建设原料基地、订单收购农产品等方式参与园区建设。推行标准化生产，落实产地准出制度，建设一批绿色农产品生产基地。支持市现代畜牧业示范区、市现代渔业示范区加快发展，争创省级农高区。大力发展农业“新六产”，建设一批田园综合体。

加快发展工厂化农业。围绕畜牧、渔业、食用菌、蔬菜、花卉、林果等主导产业，引导工商资本进入农业，支持龙头企业延伸产业链条，新上一批精深加工项目。推进澳亚高端乳制品深加工、澳源肉牛屠宰深加工等项目尽快投产，争取得利斯肉制品加工项目落地。加快海洋牧场建设，年内新建5万亩。抓好大田种植、设施农业、设施畜牧、设施渔业等数字技术示范推广，建立完善农产品质量安全追溯管理信息平台。

加强农产品品牌培育和社会化服务体系建设。以“生态黄河口、优质农产品”为主题，积极开展农产品品牌创建活动，搞好整体形象设计，统一包装推介，打造黄河口区域公用品牌，培育一批品牌产品。实施电商进村工程，加快构建农村现代流通体系，让农产品销得更远、卖得更好。积极发展农业生产性服务业，重点扶持一批规模大、可持续发展能力强的社会化服务组织。大力培育新型农业经营主体，推动农业发展方式转变和土地规模化经营。

加强水利建设。按照“灌排结合、生态改良”的要求，制定实施农田水利建设三年实施方案及年度计划。加快黄水东调工程建设。实施王庄、麻湾、胜利灌区续建配套与节水改造工程。对神仙沟、挑河、沾利河进行综合治理。加强水资源管理。大力发展节水农业和水肥一体化。

建设美丽乡村。继续提升城乡环卫一体化水平，加强农村旱厕长效管理，推进畜禽养殖废弃物无害化处理、资源化利用。继续实施天然气村村通工程，今年具备条件的村实现全覆盖。推进“四好农村路”建设，新建改造农村公路240公里，建设村道安防工程400公里，把农村公路建好、管好、护好、运营好。新建改造农村社区服务中心12处，提升规范化、标准化服务水平。搞好美丽乡村规划，突出特色，发掘和保护一批处处有历史、步步有文化的小镇和村庄。

（四）加快发展现代服务业

坚持增量与提质并重，加强引导扶持，推动生产性服务业向专业化和价值链高端提升，生活性服务业向精细化和高品质转变。大力发展旅游业，黄河口生态旅游区创建为国家5A级景区，启动孙子文化旅游区创5A和黄河水城旅游度假区创建，搞好地热资源开发利用，深入推进“厕所革命”，提升乡村旅游和工业旅游发展水平。积极发展现代物流业，加快黄河三角洲国际物流港等重点园区建设，培育一批大型物流企业；加快发展电子商务，依托优势产业，大力发展跨境电商和大宗商品电子商务，培育特色电子商务产业园区；推动批发零售业向智慧流通转变，加快线上线下融合。大力发展健康养老产业，积极探索社区嵌入式养老和农村亲情互助式养老模式，提升居家养老服务水平；加快新型养老社区建设，推进医养结合。落实房地产市场调控政策，整顿规范房地产市场秩序。优化提升商贸、餐饮、住宿、家政等传统服务业，积极发展共享经济、人力资本服务等服务业新业态。

（五）着力打造良好金融环境

银行与企业同兴衰、共命运，要像爱护自己的眼睛一样保护金融生态环境，政监银企共同努力，增进银企互信，深化银企合作。鼓励银行机构创新金融产品和服务方式，积极发展绿色金融、普惠金融，扩大信贷投放规模。推动东营银行加快上市，支持农村商业银行、村镇银行稳健发展。大力发展保险业。做大做强县域金融，更好服务“三农”和县域经济发展。健全完善金融监管体制，深化地方法人金融机构改革，引导地方金融组织规范发展。用好应急转贷资金和具有政府背景的融资担保公司，帮助企业解决资金困难。完善银行债委会工作机制，更好地发挥债委会在促进发展、防范风险中的作用。积极稳妥处置企业资金风险，运用综合手段，严厉打击恶意逃废金融债务行为。

（六）深入推进生态宜居城市建设

提升城市规划设计水平。坚持“四大”特色，启动东营市城市总体规划（2021-2035）编制工作。加快控规编制，完善城市设计体系，突出抓好北二路沿线、文化会展中心片区等重点区域、重大工程设计，努力打造一批城建精品，年内中心城实现控规和城市设计全覆盖。

加强中心城重大工程建设。加快推进国家生态园林城市创建，力争6月份达到申报条件，明年迎接现场考核验收。围绕改善生态环境，加快森林湿地公园建设，精心打造耿井水库等五湖核心区，一期工程今年“五一”对外开放，二期工程5月底前开工建设，打造中心城最具代表性的核心生态区、市民休闲区。高标准规划白鹭湿地公园，先期搞好生态保护和规划控制，逐步实施修复建设，打造湿地保护与合理利用的典范。进一步增加老城区公园、口袋公园数量，推进林荫道和绿道建设。对大清河路等3条道路实施环境改造，对华山路等9条道路实施绿化提升。今年中心城区新增绿化面积474万平方米。围绕完善城市路网，实施泸州路、温州路北延、德州路西延工程。围绕片区改造，全力推进东营河及北二路环境综合整治，尽快完成规划设计方案，全面启动道路改造、综合管廊建设、建筑景观改造和水环境治理工作；继续推进棚户区和城中村改造。对所有城建项目，严格实行工程质量终身负责制，确保质量效果，经得起检验。

深化城市精细化管理。年内，中心城环卫机械化作业率达到95%以上，道路深度保洁率达到70%以上，提升园林管护水平，深化广告牌匾等市容秩序整治，精细化管理覆盖率达到70%以上。创新城市管理和执法协调保障机制，推进城管执法网格化。继续加强拆违治乱工作，开展无违建乡镇街道、无违建县区创建活动，形成浓厚氛围和强大声势，努力实现存量和新增违法建设“双清零”。

加强县城和小城镇建设。提升县城规划建设管理水平，完善城市功能，加快建设现代化小城市。搞好大王镇新生小城市试点和省级特色小镇建设。加快推进黄河滩区旧村台改造提升，继续抓好黄河南展区新型农村社区建设。

大力推进水气土污染治理。以落实河长制为总抓手，坚持系统治理水环境，实施广利河、溢洪河及中心城内水系全流域综合治理工程，上半年基本完成中心城区的小区单位雨污分流改造，年底前其他县区城市建成区基本完成；新建、扩建、改造一批污水处理厂，确保入河水质达到五类水标准，部分达到四类水标准；建设东七路等生态湿地，提高河道水体净化能力。深入实施大气污染防治行动，全面完成燃煤锅炉超低排放改造；开展工业园区大气污染综合治理试点，实现园区污染物持续减排；优化能源结构，加快落后产能淘汰步伐；加强渣土运输车监管和建设工地管理，推广城市道路无尘作业。强化土壤污染管控和修复，加强农业面源污染防治，改善土壤质量。大力发展循环经济，分步实施垃圾分类试点，加强餐厨废弃物全过程监管，建设城市建筑垃圾处理和污泥处置设施。改革生态环境监管体制，开展市级环保督察试点。严格执行生态保护红线管理要求和污染排放标准，强化排污者责任，推行排污许可证制度，落实企业环境信用评价、严惩重罚等措施，让环境违法者付出沉重代价。

持续推进国土绿化。实施森林生态修复与保护、生态廊道和农田防护林等工程，今年新增造林6.3万亩，全面完成三年新增造林40万亩的计划目标。启动实施“绿满黄河口•美丽新东营”国土绿化行动，开展森林乡镇、森林村居创建活动，大规模增加绿化总量，提升绿化效果。强化以自然保护区、湿地公园、湿地保护小区为重点的湿地保护和恢复，狠抓中央环保督察反馈问题的整改。加强海岸带综合管理，建设美丽海岸。

（七）着力推进重大基础设施建设

铁路方面。“天津—东营—潍坊”高铁争取年底前具备开工条件，“滨州—东营—潍坊”高铁滨州至东营段争取上半年开工。加快黄大铁路、东营港疏港铁路建设和广利港疏港铁路前期工作。港口方面。加快东营港10万吨级航道建设，力争25万吨级单点系泊原油泊位年内开工。完善广利港区一期码头配套工程，启动滚装客运码头建设研究论证工作。加快推进小清河复航工作。机场方面。继续增开和优化航线航班，加快中国商飞东营基地试飞综合楼建设，全力保障C919飞机转场试飞。公路方面。加快推进长深高速广饶至高青段、庐山路南延工程，力争2019年建成通车。河广大道、南二路西延年内开工，S507董集至利津黄河大桥段改建年内完工。加快S309田高线广北农场至滨州界路段工程建设。积极推动长深高速延伸至济南东外环项目前期工作。积极推进环渤海高等级公路建设。加快实施输变电工程，提高城乡供电保障能力。

（八）深化改革扩大开放

突出重点领域改革。深化“放管服”改革，以清单管理推动减权放权，以一窗受理、一网办理推动行政审批提速增效。深入推进“证照分离”改革。深化财政体制改革，科学界定财政事权和支出责任，增强预算执行刚性约束，全面实施绩效管理。深化国资国企改革，将市属经营性国有资产全部纳入统一监管。扎实推进农村集体产权制度改革，完善市县乡三级联动的农村产权交易体系。积极推进公立医院人事薪酬制度改革，完善管理体制、价格机制、医保制度；以医联体和家庭医生签约为抓手，继续推进分级诊疗制度建设。深化校长职级制和“县管校聘”改革。加快实施文化市场综合执法改革。完成行业协会与行政机关脱钩工作。深入推进综合行政执法体制改革和事业单位分类改革。

推动形成全面开放新格局。深入实施“境外百展市场开拓计划”，办好橡胶轮胎展、石油装备展、有色金属展、石油化工展、农业博览会，加强重点产品出口基地建设和龙头企业扶持，推动对外贸易稳定增长。继续帮助企业争取进口原油使用资质。加强东营综合保税区建设运营和对外招商，积极争取设立保税区B区。加快企业“走出去”步伐，支持企业参与“一带一路”建设，引导企业加强境外资源能源战略合作，推动传统产品输出向“产品+服务”输出转型。加强国际交流合作，更好利用海外资源。

（九）推进区域协调发展

坚持油地共兴共荣，支持胜利油田加快改革、创新发展，做好“四供一业”及其他企业办社会职能接收工作；充分发挥油田科技、人才优势和地方产业、资本优势，围绕城市建设、安全环保、社会事业等领域，谋划实施一批新的共建项目。加强与中国石油大学的合作，共同推进东营校区建设发展。全力支持黄河三角洲农业高新示范区建设，集中力量加快推进重大科研创新平台和重点产业项目建设，完善管理运行体制机制，打造现代农业发展高地。支持驻地部队改革建设，深化双拥共建，促进军民融合深度发展。东营开发区，坚持高端定位，积极引进高新技术项目和央企、民营企业龙头项目，加快培育发展战略性新兴产业，在推进新旧动能转换中作出示范。东营港开发区，进一步配套完善基础设施，加快推进芳烃、乙烯、己内酰胺三大高端化工项目，打造鲁北环渤海湾高端石化基地。东营高新区，重点发展高端石油装备制造、石油工程技术服务等产业，创造条件，积极争创国家级高新区。加快发展县域经济，支持强县区争先进位，支持利津县跨越发展。

（十）提高保障和改善民生水平

优先发展教育事业。启动第三期学前教育三年行动计划，统筹推进义务教育优质均衡和城乡一体化发展，扩充普通高中优质教育资源。抓好城乡学校、幼儿园建设，加快推进市二中、市中专二期工程建设。完善现代职业教育体系，促进产教融合、校企合作。加快建设高端教育园区，积极做好招院引校工作，加快推进山东农业大学国际现代农业科教基地、北航东营飞行学院建设。加快推动山东胜利职业学院移交工作，新学期实现恢复办学，同步推进新校区规划建设。支持和规范社会力量兴办教育。加强师德师风建设，培养高素质教师队伍。开展新党校规划选址前期工作。

持续推进脱贫攻坚。扎实推进155支扶贫基金、40处扶贫关照中心、18个扶贫连片开发项目建设，落实教育、医疗、住房等保障政策，让困难群众稳定脱贫不返贫。加强困难人口动态管理，统筹解决好城镇贫困问题。

积极扩大就业创业。坚持就业优先战略和积极就业政策，提供全方位公共就业服务，加强职业技能培训，统筹抓好高校毕业生、农民工、就业困难人员等重点群体就业。加快建设各类创业示范服务平台，打造市创新创业示范基地，促进创业带动就业。

提高社会保障水平。继续提高城乡低保、农村五保供养标准。深入推进全民参保计划，强化社保基金监管，建立公共服务一体化、信息化平台。稳步提高居民医保财政补助标准，试点推进统筹城乡长期照护保险制度。加大城镇保障性住房保障力度。支持发展老龄、残疾人、慈善和社会福利事业。

加快建设“健康东营”。积极发展医疗卫生事业，培育首批健康村镇，在全省率先实现国家卫生县城全覆盖。实施基层医疗卫生机构标准化建设三年行动计划，壮大全科医生队伍，提升医疗服务水平。加强市县两级妇幼保健能力建设。稳妥推进油田医疗卫生机构移交工作，做好市级传染病医院、精神卫生中心建设前期工作。传承发展中医药事业，规划建设市中医院。加强人口计生工作，提高出生人口素质。提升食品药品检验检测能力，保障群众饮食用药安全。

繁荣发展文化体育事业。加强各级各类文化设施建设，深入实施文化惠民工程，创建成为国家公共文化服务体系示范区。实施“吕剧振兴工程”。建成启用市县综合档案馆、黄河文化馆，建设党史纪念馆。加强思想道德建设，深化群众性精神文明创建活动，提高全社会文明程度。积极发展文化产业，培育新型文化业态。广泛开展全民健身活动，提升国际马拉松、公路自行车赛办赛水平，打造耿井湖水上运动、森林湿地公园“铁人三项”等体育项目。

加强和创新社会治理。强化诚信东营建设，建成市级公共信用信息交换、共享平台和三大数据库, 建立守信联合激励和失信联合惩戒制度，让守信者一路畅通，让失信者寸步难行。深入开展“七五”普法，推进律师制度改革，完善公共法律服务体系。着力预防和化解矛盾纠纷，持续改进信访工作，加快人民调解组织专业化建设。深入推进平安东营建设三年行动，织牢织密社会治安防控网，依法打击惩治违法犯罪活动。深入开展安全生产攻坚治理，夯实安全责任，健全完善风险分级管控和隐患排查治理“两个体系”，抓好企业员工实训、危化品综合治理、安全生产综合执法等重点工作，保持安全生产形势持续稳定。

继续办好民生实事。(1)实施天然气村村通及城市建成区管道天然气全覆盖工程。(2) 实施学前教育扩容提质工程。(3) 实施扶贫济困保险惠民工程。(4) 实施智慧东营便民服务工程。(5) 实施创业就业能力提升工程。(6) 实施“全民悦读·书香东营”文化惠民工程。(7) 实施智慧医疗及基层医疗卫生服务能力提升工程。(8)建设镇村综治中心及视频监控系统。(9)建设食品安全体系。(10) 实施农村便捷出行惠民工程。

（十一）加快建设智慧东营

围绕“惠民、善政、兴业”三大目标，加强顶层设计和资源整合，统筹开发利用，加快建设智慧城市。加快互联网、大数据、云计算、物联网、人工智能与制造业深度融合，积极发展新兴产业，改造提升传统产业。整合共享城市管理信息资源，建设覆盖市县两级的数字城管系统，完善供水、供热、环卫、园林绿化等智能管理平台，促进城市管理信息化、精细化。加快编制智慧交通系统规划，建立完善交通信息交换平台，缓解交通拥堵，提升公众出行效率。启动智慧环保建设，建立覆盖全市空气质量、水环境质量、重点排污单位的监测网络，实现环境无缝隙、全覆盖、立体化监管。建立全市统一的安全生产数据中心，建设综合监管、应急救援、物联感知、综合决策平台，促进安全生产智能化、规范化。加强智慧医疗建设，建立完善人口健康信息平台，促进全市医疗卫生机构和资源的协同应用、数据共享。加强电子政务建设，提升政务服务信息化水平。积极推进其他领域的信息化工作。

创建是促进工作开展的重要抓手。今年我们要加快创建国家生态园林城市，创建国际湿地城市，创建国家全域旅游示范市，创建国家知识产权强市，创建全国质量强市示范城市，创建国家健康城市试点市，创建全国社会治安综合治理优秀市，创建省级食品安全城市，积极争创国家创新型城市，带动工作系统提升、全面上水平。

（十二）切实加强政府自身建设

要坚持从严治政。把党的政治建设摆在首位，增强“四个意识”，坚决维护以习近平同志为核心的党中央权威和集中统一领导。认真开展“不忘初心、牢记使命”主题教育，用习近平新时代中国特色社会主义思想武装头脑。全面贯彻执行上级决策部署，确保落实到位、政令畅通。持之以恒正风肃纪，凡是群众反映强烈的问题都要严肃认真对待，凡是损害群众利益的行为都要坚决纠正。巩固拓展落实中央八项规定精神成果。深化标本兼治，加强党风廉政建设，深入推进反腐败斗争。

要坚持依法行政。忠实履行宪法和法律赋予的职责，真正做到尊法学法守法用法。健全依法科学民主决策机制，广泛听取各方面意见包括批评意见。加强政府立法，严格规范公正文明执法。加强行政执法监督，做好行政复议和行政应诉工作。认真执行市人大及其常委会决定决议，做好人大代表建议和政协提案办理工作。加强政务公开和政府信息公开，让人民群众更好地监督政府。

要坚持务实从政。大兴学习之风和调查研究之风，走出去解放思想，沉下去摸透实情，增强工作本领。加强工作谋划，勇于改革创新，善于结合实际创造性推动工作。雷厉风行，提高效率，以只争朝夕的精神狠抓落实。鼓励担当，惩戒落后，旗帜鲜明为那些敢于担当、踏实做事、不谋私利的干部撑腰鼓劲，对为官不为、工作不力的严肃问责。

各位代表！新思想引领新时代，新目标开启新征程。让我们更加紧密地团结在以习近平同志为核心的党中央周围，以习近平新时代中国特色社会主义思想为指导，在省委、省政府和市委的坚强领导下，紧紧依靠全市人民，不忘初心、牢记使命，锐意进取、埋头苦干，永葆昂扬的奋斗姿态，为决胜全面建成小康社会、加快建设美丽幸福新东营而努力奋斗！
